# Gene signature to predict prognostic survival of hepatocellular carcinoma

**DOI:** 10.1515/med-2021-0405

**Published:** 2022-01-07

**Authors:** Li Li, Yundi Cao, YingRui Fan, Rong Li

**Affiliations:** Department of Oncology, The Comprehensive Cancer Centre of Drum Tower Hospital, Nanjing Drum Tower Hospital Clinical College of Nanjing Medical University, Nanjing, Jiangsu, 210000, China; Department of Medical Oncology, Affiliated Taikang Xianlin Drum Tower Hospital, Medical School of Nanjing University, Nanjing, Jiangsu, China

**Keywords:** bioinformatics, ceRNA, gene signature, TCGA, hepatocellular carcinoma

## Abstract

Hepatocellular carcinoma (HCC) has a high incidence and poor prognosis and is the second most fatal cancer, and certain HCC patients also show high heterogeneity. This study developed a prognostic model for predicting clinical outcomes of HCC. RNA and microRNA (miRNA) sequencing data of HCC were obtained from the cancer genome atlas. RNA dysregulation between HCC tumors and adjacent normal liver tissues was examined by DESeq algorithms. Survival analysis was conducted to determine the basic prognostic indicators. We identified competing endogenous RNA (ceRNA) containing 15,364 pairs of mRNA–long noncoding RNA (lncRNA). An imbalanced ceRNA network comprising 8 miRNAs, 434 mRNAs, and 81 lncRNAs was developed using hypergeometric test. Functional analysis showed that these RNAs were closely associated with biosynthesis. Notably, 53 mRNAs showed a significant prognostic correlation. The least absolute shrinkage and selection operator’s feature selection detected four characteristic genes (*SAPCD2*, *DKC1*, *CHRNA5*, and *UROD*), based on which a four-gene independent prognostic signature for HCC was constructed using Cox regression analysis. The four-gene signature could stratify samples in the training, test, and external validation sets (*p* <0.01). Five-year survival area under ROC curve (AUC) in the training and validation sets was greater than 0.74. The current prognostic gene model exhibited a high stability and accuracy in predicting the overall survival (OS) of HCC patients.

## 1 Introduction

Hepatocellular carcinoma (HCC), which is a malignant tumor originating from hepatocytes, accounts for more than 80% of all types of liver cancer. HCC is ranked as the third most common malignancy globally and is highly prevalent in Asia including in China [1]. Approximately 782,000 new HCC cases and 746,000 death cases were reported in 2012 all over the world [2]. Although surgery is a preferred treatment for HCC, most HCC patients are unsuitable for taking operation when they are diagnosed. HCC is likely to develop postoperative metastasis and recurrence, which will greatly affect the treatment outcome. Currently, the underlying molecular mechanism involved in rapid progression and high mortality of HCC is poorly understood, which points to the need to searching new molecular targets and developing new therapeutic strategies to facilitate early diagnosis and treatment of HCC and improve the overall survival of HCC patients.

Clinical treatment methods of liver cancer are surgical resection, chemotherapy, molecular targeted therapy, and liver transplantation have made encouraging progress in the past few decades [3]. However, surgery is still considered as the most effective treatment strategy to treat HCC patients. Although the overall survival rate of HCC patients has been improved, long-term survival rate remains low as >50% of HCC patients show relapses or distant metastases within 5 years; moreover, most of these patients are those previously unable to take surgical resection at the time of diagnosis [4]. Preventing a poor prognosis of liver cancer has been widely studied, and several prognostic factors (patient’s age and gender and tumor grade) have been identified to be useful in predicting the OS of patients with liver cancer [5]. Still, effective prognostic factors should be further studied.

Competing endogenous RNAs (ceRNA) are transcripts that regulate target genes at the posttranscriptional level by competitively binding to the same small RNA [6]. miRNAs are an endogenous noncoding RNAs (ncRNAs) consisting of single strands of approximately 22 nucleotides in length. miRNA–mRNA degrades or inhibits target genes through binding to MREs on target RNA transcript, thereby regulating protein expression [7]. Moreover, lncRNAs as a type of RNA with more than 200 nucleotides in length accounts for 68% of the total number of RNA molecules [8] and participate in epigenetic regulation, gene expression, and chromosome remodeling [9]. LncRNAs play an important role in the occurrence and development of tumors, but they do not code proteins. Research increasingly confirmed that disorders of lncRNAs are associated with the occurrence and development of breast cancer, lung cancer, prostate cancer, liver cancer, and other cancer types [10]. Also, lncRNAs have been found to have significant functions in tumor development mediated by ceRNA cross talking [11,12,13]. As ceRNAs, lncRNAs can enhance or suppress the inhibitory effect of miRNA on target genes and induce oncogenic or oncosuppressive genes through binding competitively to miRNA. Regulating abnormal ceRNA network results in cancer and disease development. Therefore, in-depth research on the regulation mechanism of ceRNA plays a vital role in understanding the pathogenesis of HCC.

The cancer genome atlas (TCGA), which is an open-access database containing genome-wide sequencing data sets for 33 cancers and more than 10,000 tumor samples [14], is widely used to analyze tumor-related genes, tumor pathogenesis, and tumor prognosis. Herein, we screened novel prognostic features in liver cancer through analyzing RNA-seq and miRNA expression profiles. We obtained the differentially expressed mRNAs, lncRNAs, and miRNAs data between tumor and normal samples to construct a ceRNA network. Furthermore, differentially expressed mRNAs associated with the network were selected to construct a prognostic risk model. This research discovered new biomarkers with potential prognostic value and provided preliminary bioinformatics evidence to understanding the compounded mechanisms of HCC progression.

## 2 Methods

### 2.1 Data acquisition

**Figure 1:**
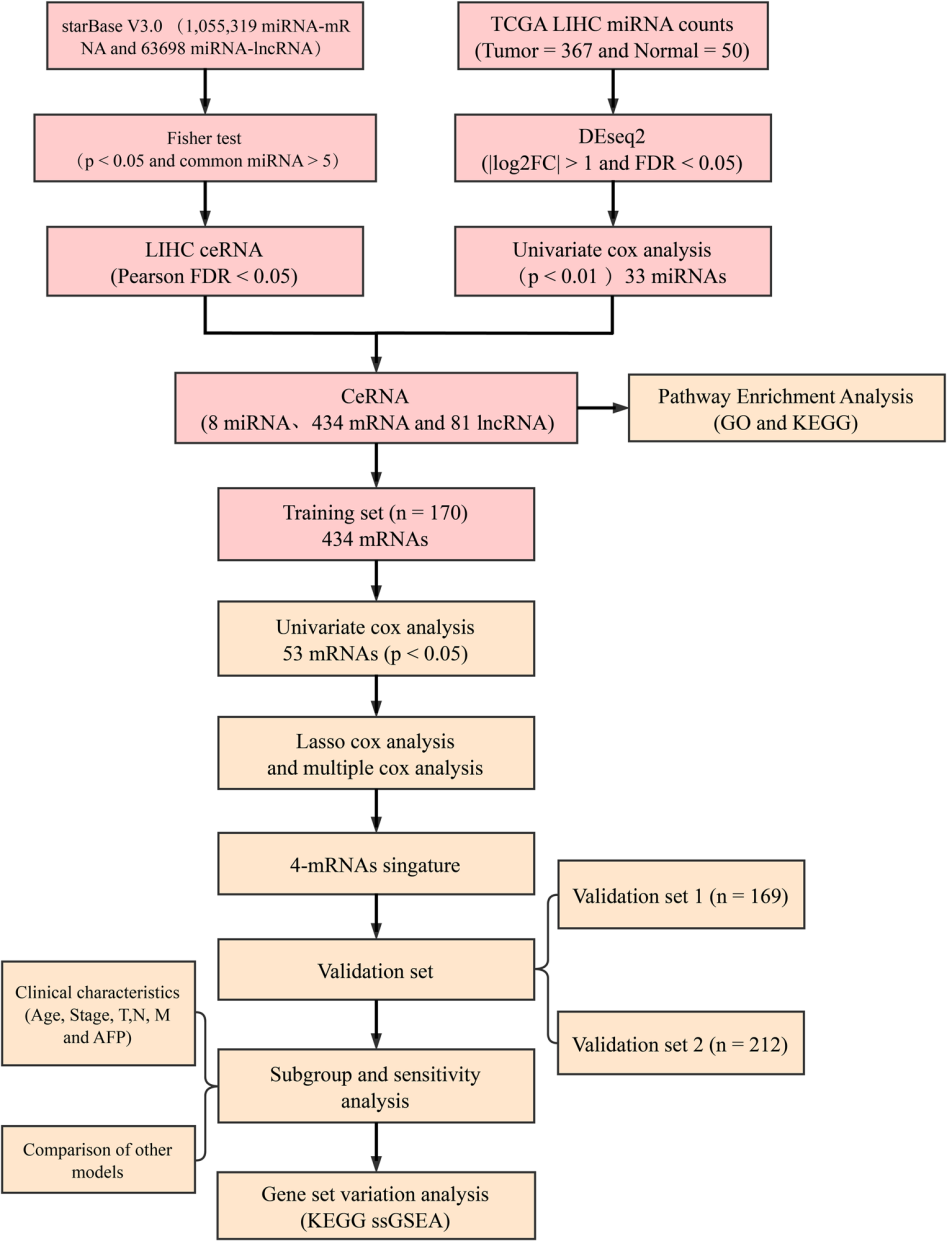
Workflow.

The RNA-seq expression (including lncRNAs and mRNAs), miRNA sequencing (miRNA-seq) data sets, and the latest clinical follow-up data were downloaded from the TCGA genomic data commons (https://gdc.cancer.gov/developers/gdc-application-programming-interface-api) on 24 January 2019. The RNA-seq data were composed of 371 liver cancer tissues and 50 adjacent tissue samples. We downloaded Count and fragments per kilobase of transcript per million fragments mapped (FPKM), and Li et al.’s method [15] was further used to convert FPKM into transcripts per million. Data on lncRNA expression profile, mRNA expression profile, and miRNA-seq from 367 liver cancer tissues and 50 paracancer tissues were extracted following the gene transfer format annotation file of the GENCODE v33 version (https://www.gencodegenes.org/human/). Moreover, international cancer genome consortium-Japan (ICGC-JP) data were obtained from the hepatocellular carcinoma database (HCCDB database (http://lifeome.net/database/hccdb/home.html), and this contained RNA-seq (including lncRNA and mRNA) and miRNA sequencing (miRNA-seq) data sets of 212 samples, and the latest clinical follow-up information. The workflow is shown in Figure 1.

### 2.2 Constructing a ceRNA network related to liver cancer gene expression

StarBase V3.0 (http://starbase.sysu.edu.cn/) integrates large-scale CLIP-seq (high-throughput sequencing of RNAs isolated by crosslinking immunoprecipitation, photoactivatable ribonucleoside-enhanced crosslinking and immunoprecipitation, individual-nucleotide resolution UV cross-linking and immunoprecipitation, UV cross-linking, ligation and sequencing of hybrids) data to decode interaction network. The miRNA target genes were predicted by five types of prediction algorithms, namely, TargetScan (http://www.targetscan.org/vert_72/), miRanda (http://miranda.org.uk/), Pictar (https://pictar.mdc-berlin.de/), PITA, and RNA22 (https://omictools.com/rna22-tool). The 1,055,319 miRNA–mRNA interaction data containing 484 miRNAs, 15,064 mRNAs, and 63,698 miRNA–lncRNA interaction data (642 miRNAs and 3,789 lncRNAs) were downloaded from starBase ev3.0. We introduced a hypergeometric model [16] to construct the ceRNA network. Briefly, the hypergeometric test was applied to calculate mRNA–lncRNA interaction based on the miRNA shared by mRNA and lncRNA as follows:

p\text{ value}=1-\mathop{\sum }\limits_{k=0}^{r-1}{\text{exp}}_{k}\times {\text{e}}_{k}^{\text{HR}}.]

More than five miRNAs were found to be shared by both mRNA and lncRNA under the selection threshold of FDR <0.05. The *p*-value was calculated as follows:

\begin{array}{c}{\text{RiskScore}}_{4}=0.2293\times {\text{exp}}^{\text{SAPCD}2}+0.3305\times {\text{exp}}^{\text{DKC}1}\\ \hspace{6em}+0.5559\times {\text{exp}}^{\text{CHRNA}5}+0.6741\times {\text{exp}}^{\text{UROD}}.\end{array}]

Finally, we calculated the correlation of the mRNA–lncRNA pairs in the expression profile of liver cancer samples, and correlation coefficient greater than 0 and FDR <0.05 were selected to construct a global network of liver cancer ceRNA.

### 2.3 Identifying miRNA imbalance-mediated ceRNA networks

The R package DESeq2 [17] (Department of Biostatistics and Computational Biology, Dana Farber Cancer Institute and Department of Biostatistics, Harvard School of Public Health, MA USA) was used to identify differential miRNAs. After eliminating mRNAs with an average count of less than five from the expression profile, we compared the differences between tumor and normal samples with the threshold of |log 2(foldchange)| >1 and false discovery rate (FDR) <0.05. Furthermore, the correlation between the differential miRNA expressions and the prognosis was analyzed using univariate Cox analysis with *p* <0.05. The prognosis-related differential miRNAs were selected and mapped into the global HCC network of ceRNA to extract subnets and determine the miRNA imbalance-mediated ceRNA network.

### 2.4 Constructing the prognostic gene signature

In this experiment, we obtained the TCGA dataset and selected samples with a follow-up time of longer than 30 days. The TCGA data were grouped at 1:1 ratio. The training set and TCGA test set contained 170 and 169 samples, respectively. The ICGC data set served as an external validation set (*N* = 212). The clinical characteristics of the three data sets are shown in Table 1. Genes in the ceRNA network mediated by miRNA misregulation were used as candidate features. Correlation of gene expression with prognosis of samples in the TCGA training set was analyzed through performing univariate Cox analysis (significant at *p* <0.01). The least absolute shrinkage and selection operator (Lasso) Tibshirani (1996) method was used to further reduce the gene range. Thereafter, a prognostic model based on these genes was established. The R software package glmnet [18] (Division for Infection Control and Environmental Health, Department of Infectious Disease Epidemiology and Modelling, Norwegian Institute of Public Health, Oslo, Norway) was used for Lasso Cox regression analysis. Furthermore, a 10-fold cross-validation was applied for the model development. The optimal lambda was selected according to the change trajectory of each independent variable. Finally, stable potential prognostic indicators were determined, and gene combination with the smallest akaike information criterion (AIC) was identified to be the final prognostic markers using the stepwise multifactor Cox regression method. The formula for the risk score was as follows:

\begin{array}{c}{\text{RiskScore}}_{4}=0.2293\times {\text{exp}}^{\text{SAPCD}2}+0.3305\times {\text{exp}}^{\text{DKC}1}\\ \hspace{6em}+0.5559\times {\text{exp}}^{\text{CHRNA}5}+0.6741\times {\text{exp}}^{\text{UROD}}.\end{array}]

**Table 1:** TCGA and ICGC sample statistics

Characteristic	Training set (*n* = 170)	Testing set (*n* = 169)	*p*-value	ICGC set (*n* = 212)
Age (years)				
≤60	81	82	0.958	43
>60	89	87		169
Survival status				
Living	110	109	1	176
Dead	60	60		36
Gender				
Female	46	61	0.094	50
Male	124	108		162
Grade				
G 1	21	32	0.419	—
G 2	82	76		—
G 3	59	53		—
G 4	6	6		—
Pathologic_T				
T 1	81	85	0.665	—
T 2	39	44		—
T 3	40	34		—
T 4	8	5		—
Pathologic_N				
N 0	111	126	0.096	—
N 1	2	1		—
N *X*	56	42		—
Pathologic_M				
M 0	119	124	0.569	—
M 1/M *X*	51	45		—
Tumor stage				
Stage I	77	82	0.599	33
Stage II	34	42		102
Stage III	43	36		61
Stage IV	2	1		16
AFP				
AFP >300	28	34	0.424	—
AFP ≤300	102	94		—
Race				
White	84	81	0.519	—
Asian	69	79		—

### 2.5 Functional enrichment analysis

Gene ontology (GO) and Kyoto Encyclopedia of Genes and Genomes (KEGG) pathway enrichment analyses were conducted in the R package clusterProfiler [19] to identify overrepresented GO terms in three categories (biological processes, molecular function, and cellular component) and KEGG pathway (FDR  < 0.05 denoted statistical significance). gene set variation analysis [20] was conducted on C2 canonical pathway gene set collection containing 1,320 gene sets in the R package using the molecular signatures database [21]. We used the single-sample gene set enrichment analysis (GSEA) to analyze enrichment score of each sample in the gene sets [20,22]. Pearson rank correlation coefficient was used to calculate the correlation between the enrichment score of each gene sets and RiskScore and KEGG pathways of an absolute correlation coefficient >0.5 and FDR <0.01.

### 2.6 Statistical analysis

All analyses were performed in R packages 3.4.3 using default parameters unless otherwise stated. Student’s *t*-test and two-sided tests were used for statistical tests. Pearson correlation coefficient was applied for correlation analysis. Cytoscape [23] (http://www.cytoscape.org/) was conducted for network visualization. The Benjamini–Hochberg method was used to convert the *p*-value to FDR. Survival curves for each subgroup in the data set was plotted by The Kaplan–Meier method. Additionally, the log-rank test was used to determine statistically significant differences at *p* <0.05.

## 3 Results

### 3.1 Constructing HCC-specific ceRNA network and mRNA–lncRNA coexpression characteristics

**Figure 2:**
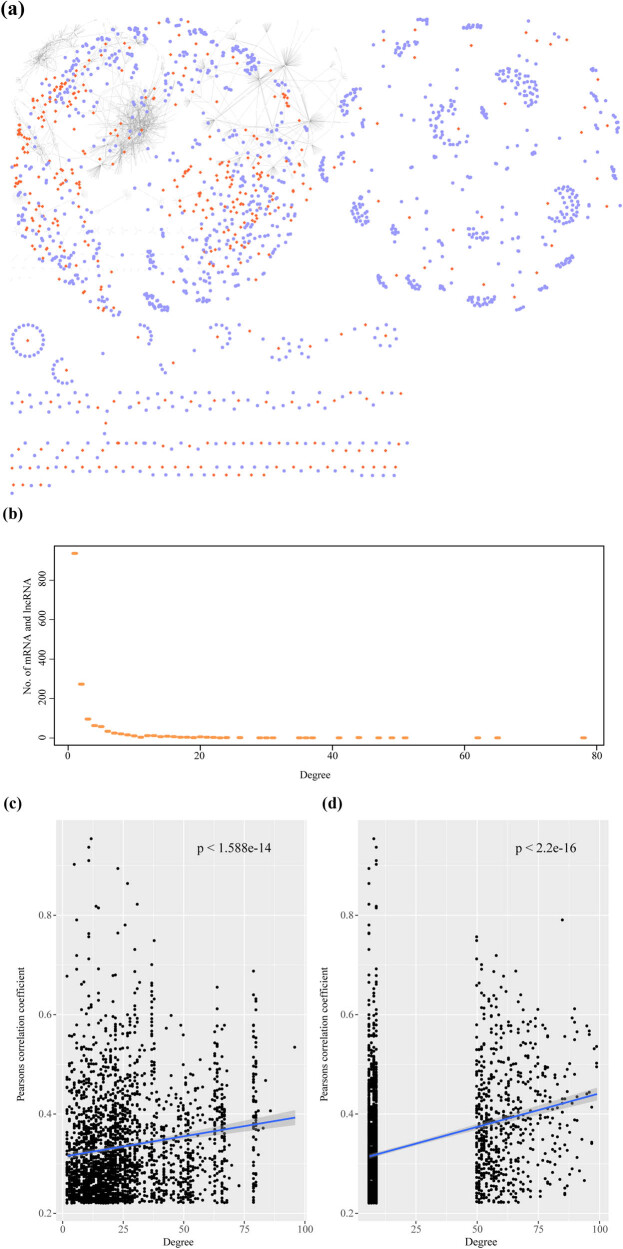
Constructing HCC-specific ceRNA network and mRNA–lncRNA coexpression characteristics: (a) ceRNA network in the HCC sample. The blue node in the figure represents the gene, and the orange-red node represents the lncRNA, (b) networks degree distribution of ceRNA in HCC samples, (c) correlation between the network degree of ceRNA and coexpression of mRNA–lncRNA in HCC samples, and (d) correlation between the number of miRNAs shared by mRNA–lncRNA and the coexpression of mRNA–lncRNA in the ceRNA network for HCC samples.

We generated ceRNA landscapes of HCC samples based on expression profiles to systematically evaluate the potential role of miRNA-mediated ceRNA networks in HCC. First, 3,112 genes associated with the 15,364 miRNA-mediated mRNA–lncRNA pairs were screened by the hypergeometric test. Second, the correlation of the mRNA–lncRNA pairs in the expression profile of HCC samples was calculated to construct an HCC sample-specific ceRNA network. The ceRNA network was determined with a correlation coefficient >0 and an FDR <0.05. Finally, 2,526 mRNA–lncRNA pairs of 1,227 genes and 400 lncRNAs were identified (Figure 2a). One lncRNA tended to form a ceRNA network with multiple genes, and the degree distribution showed a power-law distribution. This was consistent with the characteristics of biological networks (Figure 2b), indicating that ceRNA was potentially associated with liver-regulated networks in HCC. Through network topological property analysis, we found that the degree in the network was significantly positively correlated with mRNA–lncRNA expression. The genes with a high network degree demonstrated higher expression correlation coefficients (Figure 2c). Also, mRNA–lncRNA interactions shared more miRNAs with higher correlation coefficients (Figure 2d).

### 3.2 Identifying dysregulated ceRNA and functional enrichment analysis

**Figure 3:**
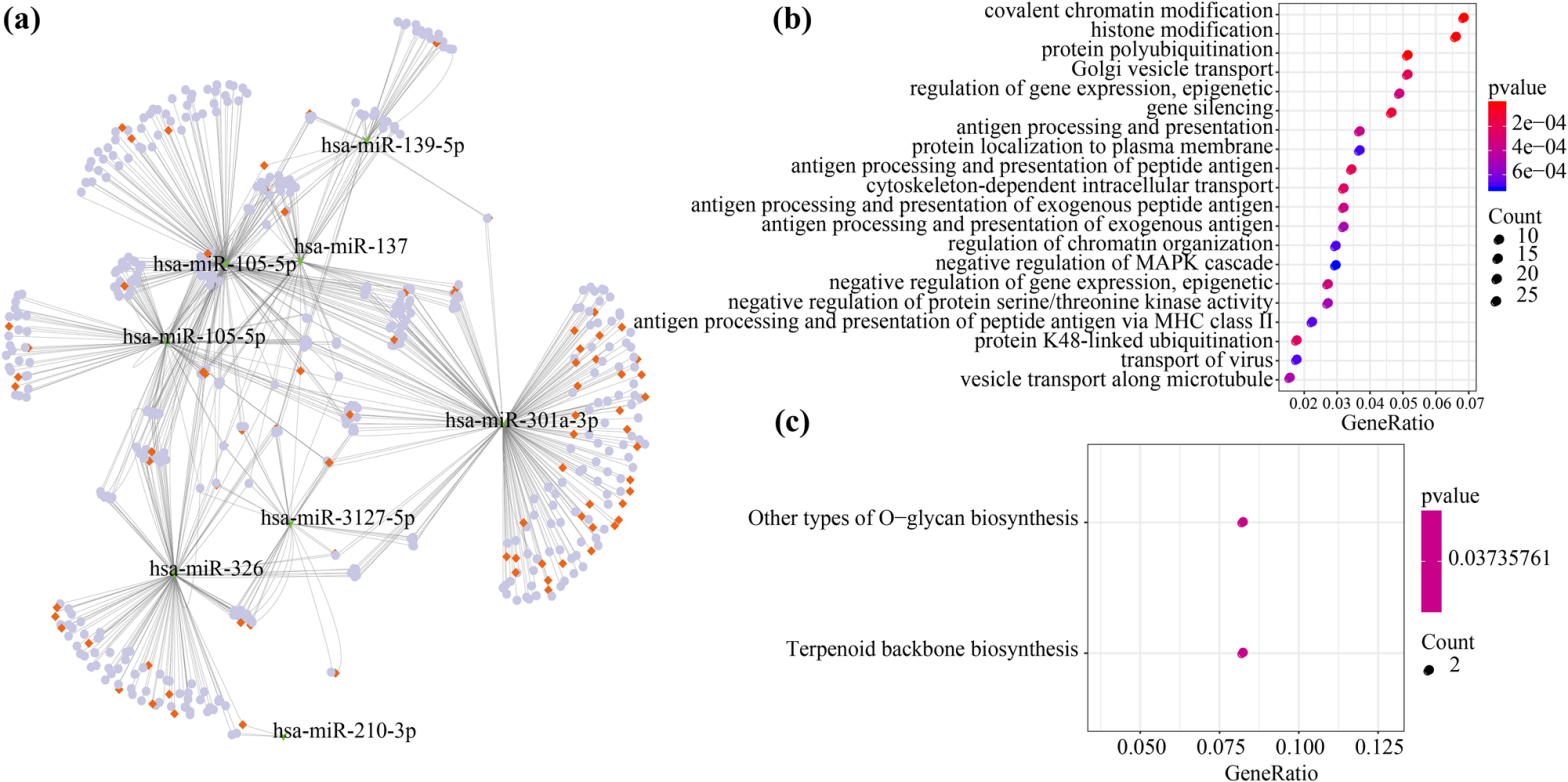
Identifying dysregulated ceRNA and functional enrichment analysis: (a) the disordered ceRNA network in liver cancer, where the orange block represents the lncRNA, the green arrow represents the miRNA, and the light blue circle represents the mRNA; (b) the top 20 most significant results of 434 mRNA GO term enrichment in ceRNA network; and (c) the 434 mRNAs in the ceRNA network are enriched in two KEGG pathways, in which the color represents a significant *p*-value, the smaller the *p*-value, the darker the color, the size of the circle represents the number of genes in the enrichment pathway, and the larger the circle, the more the number of genes.

We first identified 261 differential miRNAs between liver cancer samples and normal samples. Notably, 33 miRNAs were found to be significantly associated with HCC prognosis by univariate Cox analysis. Then, the prognostic and dysregulated miRNA-mediated mRNA–lncRNA pairs were identified and mapped into the HCC-specific ceRNA network. Here, a total of 769 pairs of miRNA–lncRNA, 8 miRNAs, 434 mRNAs, and 81 lncRNAs were obtained (Figure 3a). Functional enrichment analysis was performed on 434 differentially expressed genes in the ceRNA network, which contributed to the understanding of the functional implications of the ceRNA network. Moreover, GO and KEGG enrichment analyses were conducted using clusterProfiler in R, and the genes were enriched to 83 GO terms and 2 KEGG pathways (Figure 3b and c). Among the genes, GO pathway was mainly associated with gene silencing, negative regulation of mitogen-activated protein kinases cascade, negative regulation of gene expression, epigenetic, and some other processes, and the KEGG pathway was mainly associated with other types of O-glycan biosynthesis and terpenoid regular biosynthesis pathways. Many reports have shown that abnormal tumor proliferation could cause abnormal expression of enzymes during biosynthesis [24,25].

### 3.3 Identifying ceRNA associated four-gene signature for HCC survival

**Figure 4:**
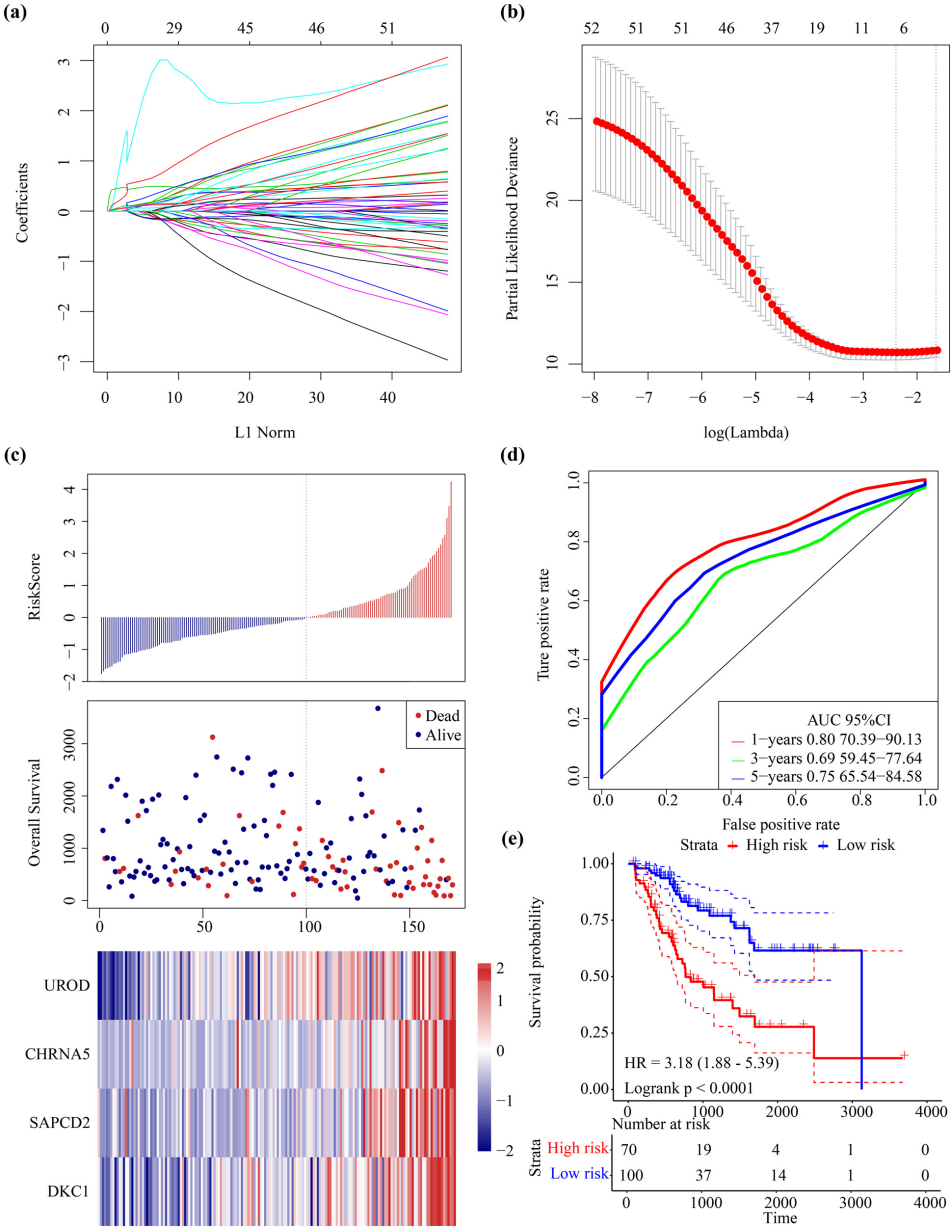
Identifying ceRNA associated four-gene signature for HCC survival: (a) the trajectory for each independent variable. The horizontal axis represents the log value of the independent variable lambda, and the vertical axis represents the coefficient of the independent variable, (b) the confidence interval for each lambda, (c) risk score, survival time and survival status, and expressions of 4-mRNAs in TCGA training set, (d) ROC curve and AUC of the 4-mRNAs signature, and (e) KM survival curves distribution of 4-mRNA signature in the TCGA training set.

**Figure S1:**
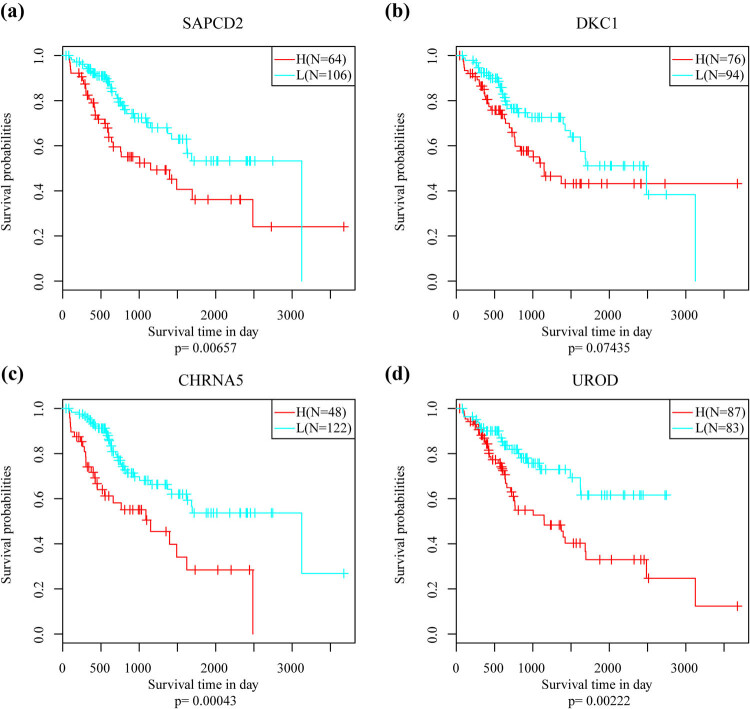
KM OS curve of (a) SAPCD2, (b) DKC1, (c) CHRNA5 and (d) UROD in high and low risk groups.

In the TCGA training set samples, 434 candidate differentially expressed mRNAs in the ceRNA network were first analyzed by univariate Cox analysis to determine whether the three mRNAs were significantly associated with HCC prognosis. We further narrowed the gene range and constructed a highly accurante prognosis model based on the 53 mRNAs. The R software package glmnet was used for Lasso Cox regression analysis. First, after analyzing the change trajectory of each independent variable, it was observed that the number of independent variable coefficients close to 0 gradually increased with a gradual increase of lambda (Figure 4a). The model was constructed with 10-fold cross-validation, and the confidence interval under each lambda was analyzed. As the model was considered to be optimal where at lambda = 0.0914 (Figure 4b), according to which seven genes were determined to be target genes and further analyzed using multivariate Cox survival analysis. Four mRNAs with the minimum AIC value (AIC = 500.30) were retained in the final model (Table 2). All the four genes could effectively divide TCGA training set samples into high-risk and low-risk groups (Figure S1). The multivariate Cox proportional risk regression model was used to assess the relative weight of the genes in the risk score. The formula was as follows:

\begin{array}{c}{\text{RiskScore}}_{4}=0.2293\times {\text{exp}}^{\text{SAPCD}2}+0.3305\times {\text{exp}}^{\text{DKC}1}\\ \hspace{6em}+0.5559\times {\text{exp}}^{\text{CHRNA}5}+0.6741\times {\text{exp}}^{\text{UROD}}.\end{array}]

HCC patients were divided into two groups based on the median risk score. The survival time of the dead samples was significantly shorter if the risk score became higher and accounted for the majority of the high-risk group. Moreover, the expression value of four different signature mRNAs increased with an increase of the risk score, which indicated that high expression of the four mRNAs was associated with a higher risk and were, therefore, considered as risk factors (Figure 4c). The predictive performance of the prognostic signature for 1, 3, and 5 years was evaluated using time-dependent receiver operating characteristic (ROC) and showed an average area under ROC curve (AUC) of 0.75 (Figure 4d). Significant differences between the high-risk and low-risk groups were observed using prognostic survival analysis (Figure 4e).

### 3.4 Robustness of the 4-mRNA signature

**Figure 5:**
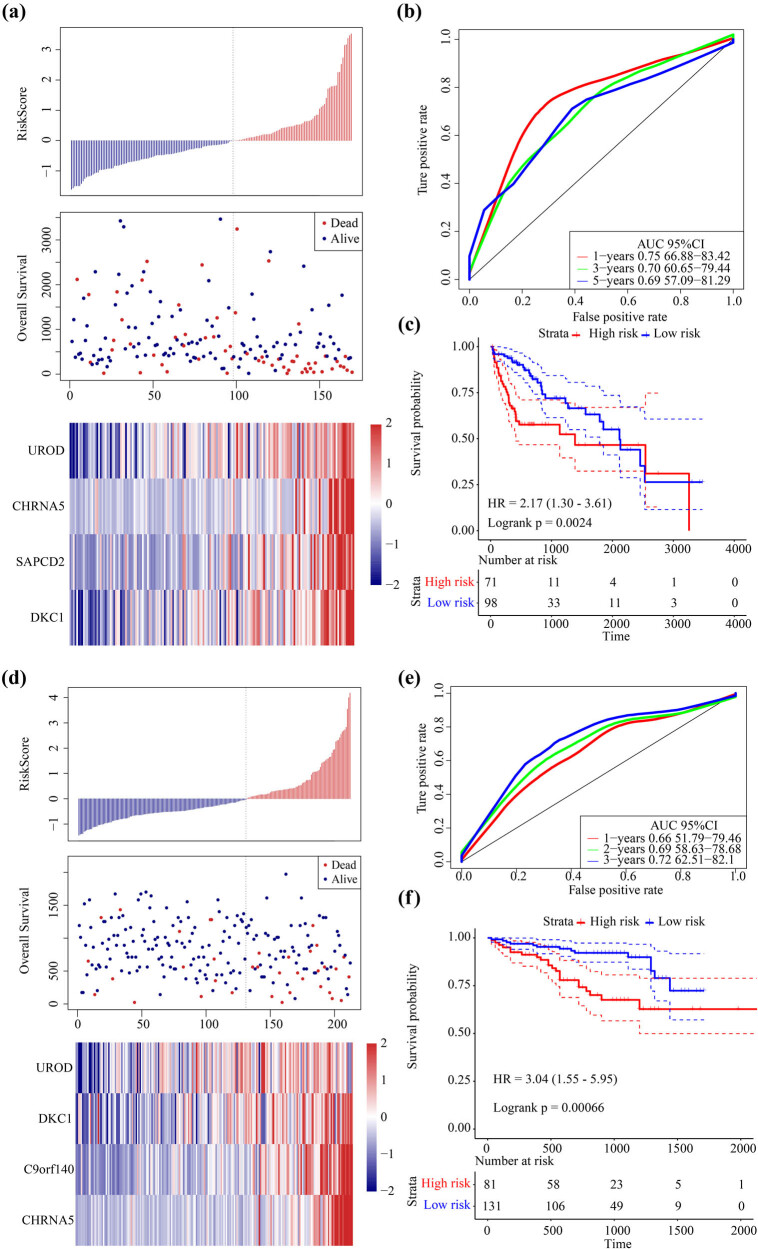
Robustness of 4-mRNA signature: (a) risk score, survival time and survival status, and expressions of 4-mRNAs in TCGA test set; (b) ROC curve and AUC of the 4-mRNA signature; (c) KM survival curves distribution of 4-mRNA signature in the TCGA test set; (d) the risk score, survival time and survival status, and expressions of 4 mRNAs in ICGC data set; (e) ROC curve and AUC of the 4-mRNA signature; and (f) KM survival curve distribution of 4-mRNA signature in the ICGC data set.

The same model used for TCGA validation set was applied to the external independent data set ICGC data set. High-risk scores were found to correspond to higher death and gene expression in the samples from both datasets (Figure 5a and d). ROC analysis showed that the average AUC for 1, 3, and 5 year was 0.71 in the TCGA testing set and 0.69 in the ICGC data set (Figure 5b and e). Prognostic survival analysis showed significant differences between high-risk and low-risk groups in the TCGA test set and ICGC data set (Figure 5c and f).

### 3.5 Association of 4-mRNA signature and clinical characteristics

**Figure 6:**
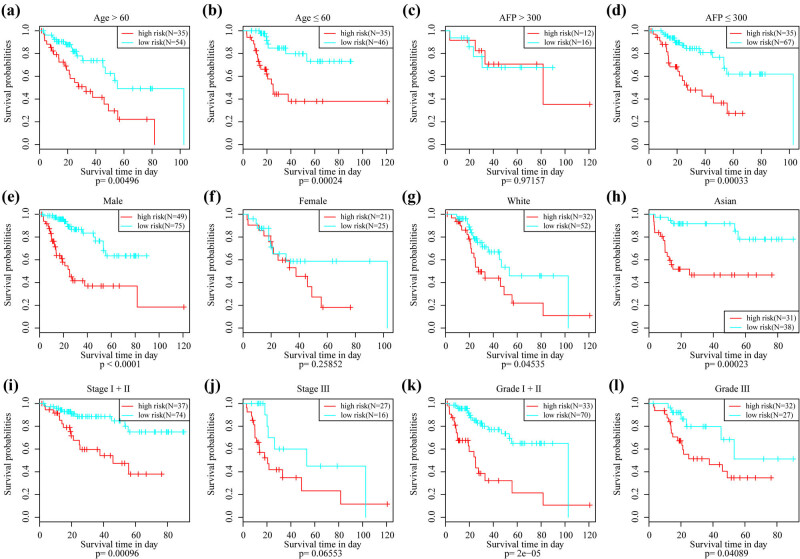
KM curve analysis of 4-mRNA signature in clinical features: (a) prognostic KM curve in elderly samples (age >60), (b) prognostic KM curve in young samples (age ≤60), (c) prognostic KM curve in serum AFP greater than 300 samples, (d) prognostic KM curve in serum AFP <300 samples, (e) prognostic KM curve in the male sample, (f) prognostic KM curve in the female sample, (g) prognostic KM curve in Caucasian samples, (h) prognostic KM curve in Asian samples, (i) prognostic KM curve in Stage I + Phase II samples, (j) prognostic KM curve in Stage III samples, (k) prognostic KM curve in Grade I + II samples, and (l) prognostic KM curve in Grade III samples.

**Figure S2:**
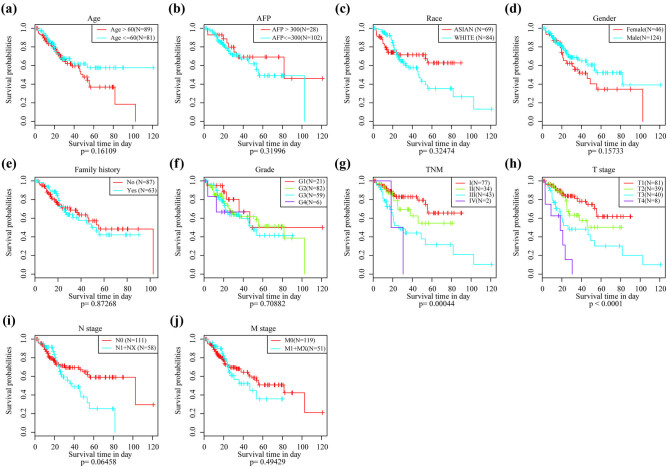
KM curve of (a) Age, (b) AFP, (c) Race, (d) Gender, (e) Family history, (f) Grade, (g) TNM phase, (h) T phase, (i) N phase and (j) M phase in HCC OS in the TCGA training set.

From the results of survival analysis, only the tumor-node-metastasis (TNM) and tumor phases were found to be significantly correlated with the OS of HCC in the TCGA training set (Figure S2). A subgroup analysis of the four-gene signature revealed that 4-mRNA signature could significantly distinguish young, old, AFP ≤300, male, white, Asian, Stage I + II, grade I +, and grade III patients from high-risk and low-risk groups (Figure 6). Patients with Stage III showed significant margins (*p* = 0.065) while those with AFP > 300 showed no significant margins, which may be explained by limited sample size. Relevant HR, 95% CI of HR were analyzed, and the *p*-value using univariate and multivariate Cox regression on the TCGA and ICGC data to determine whether the 4-mRNA signature model can be independently used in clinical applications. Clinical information on TCGA and ICGC patient records including age, gender, pathology T phase, node phase, metastasis phase, tumor stage, and our 4-mRNA signature grouping information were systematically analyzed (Table 3). In the TCGA dataset, univariate Cox regression analysis showed that N-stage, M-segment, Stage III/IV vs Stage I/II, and risk score were significantly associated with patients’ survival. However, the corresponding multivariate Cox regression analysis demonstrated that the risk score (HR = 2.558, 95% CI = 1.673–3.913, *p* = 1.47 × 10^−5^), and M stage were significantly associated with survival. In the ICGC dataset, univariate Cox regression analysis revealed that the risk score and Stage III/IV vs Stage I/II were significantly associated with survival. Also, from the data of the corresponding multivariate Cox regression analysis, it was found that the risk score (HR = 2.984, 95% CI = 1.506–5.913, *p* = 0.0017), gender, and Stage III/IV vs Stage I/II were significantly related to HCC survival. The above results indicated that our 4-mRNA signature model had a high performance in clinical prediction of HCC survival.

**Table 2:** 4-mRNA signature

Symbol	Coef	HR	*Z*-score	*p*-value	Low 95% CI	High 95% CI
SAPCD2	0.2293	1.258	1.662	0.09657	0.9597	1.648
DKC1	0.3305	1.392	1.782	0.07476	0.9675	2.002
CHRNA5	0.5559	1.743	2.276	0.02284	1.0803	2.814
UROD	0.6741	1.962	2.916	0.00354	1.2474	3.087

**Table 3:** Univariate and multivariate COX regression analyses identify clinical factors associated with prognosis

Variables	Univariate analysis			Multivariable analysis		
	HR	95% CI of HR	*p-*value	HR	95% CI of HR	*p-*value
Entire TCGA cohort						
4-mRNA risk score						
Risk score (high/low)	2.684	1.865–3.864	1.07 × 10^−7^	2.558	1.673–3.913	1.47 × 10^−5^
Age	1.008	0.994–1.022	0.252	1.005	0.989–1.021	0.541
Gender (male/female)	0.804	0.556–1.163	0.247	0.812	0.541–1.218	0.314
AFP	1.067	0.642–1.775	0.801	—	—	—
T3/T4 vs T1/T2	2.901	2.016–4.176	9.90 × 10^−9^	2.791	0.357–21.833	0.328
N1/N2 vs N0	1.559	1.066–2.280	0.022	1.053	0.629–1.763	0.845
M1/MX vs M0	1.732	1.184–2.533	0.005	1.814	1.104–2.979	0.019
Stage III/IV vs stage I/II	2.827	1.923–4.154	1.23 × 10^−7^	0.922	0.119–7.165	0.938
G3/G4 vs G1/G2	1.085	0.745–1.578	0.671	0.946	0.612–1.462	0.802
ICGA cohort						
4-mRNA risk score						
Risk score (high/low)	3.038	1.551–5.949	0.0012	2.984	1.506–5.913	0.0017
Age	1.015	0.979–1.052	0.406	1.007	0.971–1.045	0.691
Gender (male/female)	0.516	0.256–1.039	0.064	0.337	0.156–0.733	0.006
Stage III/IV vs stage I/II	2.737	1.415–5.295	0.0028	3.282	1.624–6.632	0.0009

### 3.6 Comparing the 4-mRNA signature with the existing model and potentially related regulatory pathways

**Figure 7:**
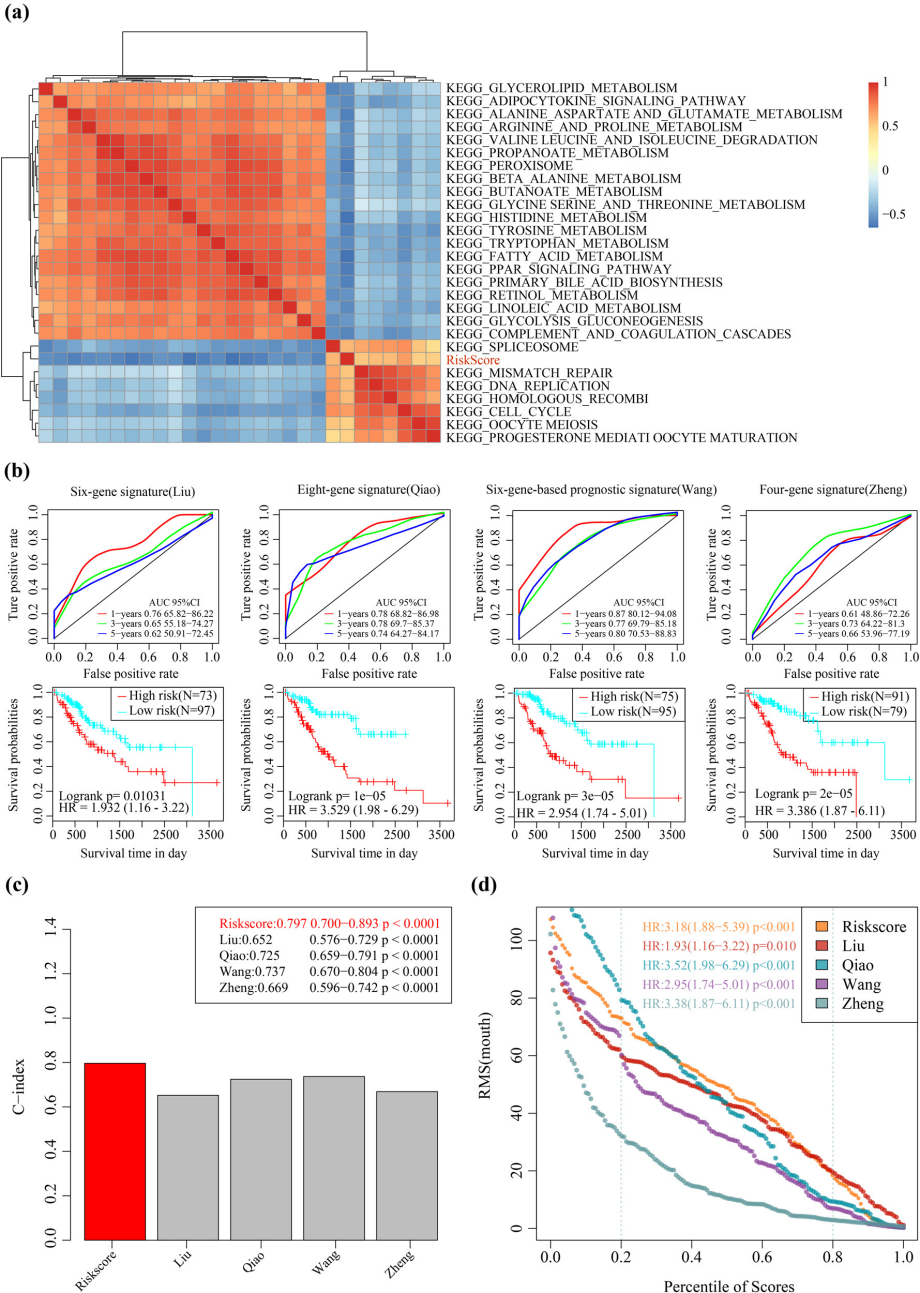
Comparing the 4-mRNA signature with the existing model and the potential-related regulatory pathway: (a) clustering of correlation coefficients between KEGG pathways with RiskScore greater than 0.5 and RiskScore, (b) AUC curve and prognosis KM curve of four models in the TCGA training set, (c) C-index of five prognostic risk models, and (d) restricted mean survival (RMS) curve of five prognostic risk models. The dashed line represents RMS time (months) corresponding to 20 and 80% percentile score, respectively.

The correlation of the risk score in different samples with the biological functions was determined. We obtained the single-sample GSEA score corresponding to each sample through using different function calculations, and the correlation of these functions with the risk score was also analyzed. Function with a correlation value greater than 0.5 and FDR <0.01 was filtered (Figure 7a). Notably, 20 pathways were negatively correlated with the risk score, whereas 7 pathways were positively correlated with the risk score. Cell division and proliferation-related pathways such as cell cycle and DNA replication were the major positively related pathways, suggesting that cell cycle and DNA repair were abnormally active in high-risk samples. Additionally, negatively related pathways such as glycerolipid metabolism and histidine metabolism were found to be mainly associated with metabolism, suggesting that the imbalance of these pathways may cause tumors.

Four prognostic risk models including six-gene signature (Liu et al.) [26], eight-gene signature (Qiao et al.) [27], six-gene-based prognostic signature (Wang et al.) [28], and four-gene signature (Zheng et al.) [29] were selected for comparison with our four-gene model. In this experiment, the risk score of each liver hepatocellular carcinoma sample in TCGA was calculated using the same method. Then, we analyzed the ROC of each model. Note that among the four models, the six-gene-based prognostic signature (Wang) was similar to the 4-mRNA signature, whereas the other three models had lower AUC than the 4-mRNA signature. The prognosis in high-risk group was significantly worse compared with the low-risk group in the four models (Figure 7b). Furthermore, comparison of c-index of the four models and 4-mRNA signature showed that the 4-mRNA signature had the highest c-index (Figure 7c). Analysis on restricted mean survival showed that the 4-mRNA signature and eight-gene signature (Qiao) were highly accurate in predicting the long-term HCC survival (Figure 7d).

## 4 Discussion

In this study, we obtained lncRNA, mRNA, and miRNA expression profiles from the TCGA database and developed a ceRNA network for HCC, based on which 53 differentially expressed mRNAs were identified to be able to independently predict OS of HCC patients. Notably, comprehensive analysis on 53 mRNAs using LASSO regression generated four a gene-based signature (*SAPCD2*, *DKC1*, *CHRNA5*, and *UROD*) related to the OS of HCC. Furthermore, the signature was verified as an independent indicator using internal data sets and external validation queues. Functional analysis showed that risk score values were correlated with cell cycle, DNA replication, glycerolipid metabolism, and histidine metabolism.

Moreover, the ceRNA hypothesis is a novel regulatory mechanism that functions through miRNA competition [6]. Studies have used the TCGA database to assess ceRNA networks of several cancer types, including HCC [30], lung cancer [31], and gastric cancer [32]. Zhang et al. identified a ceRNA network using the TCGA HCC dataset [30]. Wang et al. [33] proposed highly upregulated in liver cancer as a mechanism for ceRNA to participate in the ceRNA regulation network. Overexpressed linc00974 has been found to interact with has-mir-642a-5p to upregulate the gene expression of KRT19, which further activates Notch and transforming growth factor-beta signaling pathways and enhances proliferation and invasion ability of HCC. Thus, linc00974 is closely associated with HCC progression [13]. Moreover, Yan et al. applied TCGA data and identified a circRNA-microRNA-mRNA regulatory network in HCC [34]. Herein, we set the differentially expressed RNA at |log FC| >1 and FDR <0.05, and established a prognostic signature as a potential independent indicator of OS in HCC based on the RNAs from the ceRNA network.

The prognostic markers included four differentially expressed genes (DEGs) (SAPCD2, DKC1, CHRNA5, and UROD). However, previous studies have reported the prognostic significance of DEGs in cancer and their dysregulation in tumor tissues. Moreover, studies reported that DKC1 upregulation is frequently observed in many different human cancers including in HCC, and HCC tissues show a positive association with nuclear DKC1 levels [35]. Additionally, DKC1 overexpression in HCC patients was correlated with an advanced clinical stage and poor prognosis [36]. The remaining three DEGs (SAPCD2, CHRNA5, and UROD) have rarely been reported in HCC. The mRNA and protein level of SAPCD2 was found to be upregulated in nasopharyngeal carcinoma tissues, and gain-of-function and loss-of-function experiments demonstrated that SAPCD2 could promote nasopharyngeal carcinoma cell proliferation, migration, and invasion [37]. In addition, previous study using RNA-seq analysis found SAPCD2 nonsynonymous single-nucleotide polymorphisms in five pairs of lung adenocarcinoma tumor tissues but not in any other adjacent normal tissues [38]. CpG site-annotated genes such as *SAPCD2* are critical in the progression of cancers and cardiovascular diseases [39]. A previous report suggested that CHRNA5 RNAi was associated with cell cycle inhibition, apoptosis, reduced DNA damage response, and increased drug sensitivity in breast cancer [40]. CHRNA5 (15q25.1) is localized in lung cancer susceptibility loci, and CHRNA5 polymorphism is related to lung cancer susceptibility [41,42,43]. However, UROD has not been reported in cancer.

Despite the novel findings, here we reported some limitations of this study. First, the samples lacked clinical follow-up information; therefore, factors such as other health statuses of patients were not included in distinguishing clinical outcomes. Second, the results obtained by bioinformatics analysis may not be convincing enough and require experimental verification. Therefore, further genetic and experimental studies incorporating larger sample sizes and experimental validation are needed.

This study identified several differentially expressed genes, miRNAs, and lncRNAs from primary HCC tumors and adjacent normal liver tissues. A ceRNA network for HCC was established based on the maladjusted RNAs, and a prognostic signature for predicting the OS of HCC patients was developed. The current findings highlighted the underlying mechanism through which dysregulated RNAs participate in the development and prognosis of HCC. The results of this study provide new insights into the development of novel clinical diagnostic and therapeutic biomarkers for HCC.
